# Linbots: Soft Modular Robots Utilizing Voice Coils

**DOI:** 10.1089/soro.2018.0058

**Published:** 2019-04-16

**Authors:** Ross M. McKenzie, Mohammed E. Sayed, Markus P. Nemitz, Brian W. Flynn, Adam A. Stokes

**Affiliations:** ^1^ Scottish Microelectronics Centre, School of Engineering, Institute for Integrated Micro and Nano Systems, The University of Edinburgh, Edinburgh, United Kingdom.; ^2^ EPSRC CDT in Robotics and Autonomous Systems, Edinburgh Centre for Robotics, Edinburgh, United Kingdom.; ^3^ Department of Computer Science and Engineering, University of Michigan, Ann Arbor, Michigan.

**Keywords:** soft, modular, voice coils, Linbots, robots, soft robotics

## Abstract

Robots performing automated tasks in uncontrolled environments need to adapt to environmental changes. Through building large collectives of robots, this robust and adaptive behavior can emerge from simple individual rules. These collectives can also be reconfigured, allowing for adaption to new tasks. Larger collectives are more robust and more capable, but the size of existing collectives is limited by the cost of individual units. In this article, we present a soft, modular robot that we have explicitly designed for manufacturability: Linbots. Linbots use multifunctional voice coils to actuate linearly, to produce audio output, and to sense touch. When used in collectives, the Linbots can communicate with neighboring Linbots allowing for isolated behavior as well as the propagation of information throughout a collective. We demonstrate that these collectives of Linbots can perform complex tasks in a scalable distributed manner, and we show transport of objects by collective peristalsis and sorting of objects by a two-dimensional array of Linbots.

## Introduction

### Large robotic collectives allow for robust behavior

There has been major interest in modular robotic systems over the past years. This interest is due to the hypothesis that multiple low-cost units are more robust than a single, advanced robot.^1,2^ Modular robot collectives can be reconfigured for different tasks,^1,3–5^ setting them apart from monolithic robots. Collectives of modular robots have greater capability than the sum of their parts.^6^ This increased capability allows modular collectives to be used for tasks that are not possible with monolithic robots such as reconfigurable furniture^7^ and modeling cell collectives.^8^ Although modular actuators can also be combined into a single system for tasks such as controllable surfaces,^9–11^ collectives of modular robots have the advantages of reconfigurability and inbuilt sensing and computation.

The capability of a modular robot collective is dependent on the number of units within it.^1^ The size of a collective robotic system, however, is often limited by cost, with larger groups sacrificing the functionality of individual robots to keep costs low. Creating low-cost robots with more capabilities is one of the key challenges in collective robotics. In this work, we developed modular, soft robots, demonstrated in Supplementary Video S1 designed for manufacturability and which use multifunctional components to reduce hardware costs.

### Multifunctional hardware can lower costs

The use of multifunctional hardware is demonstrated by the Kilobot.^12^ The Kilobot is the lowest cost (∼$15) robot currently available. It uses an actuation mechanism based on cheap vibrational motors. It also uses a single infared radiation (IR) sensor for both proximity sensing and communication. Through pulsing its IR transmitter, a Kilobot is able to communicate with others and by measuring the intensity of incoming IR communications. It is also capable of detecting how close other Kilobots are to it. This use of a single piece of hardware for multiple functionalities reduces the cost of robots.

Building a robot with an extensible architecture allows extra sensors to be easily added to the robot.^13^ This architecture allows the user to define the required abilities of each robot and keeps the cost-to-volume ratio balanced with respect to the task at hand. The base of the robot can then be repurposed for different tasks.

The HoverBot^14^ uses a Hall-effect sensor for both odometry and navigation.^15^ This swarm robot is made of a single printed circuit board (PCB) with electromagnetic coils and uses an air table with embedded permanent magnets for low-cost locomotion. Absent magnets on the air table create magnetic landmarks that can be detected by the HoverBots. This dual use of a Hall-effect sensor allows the number of required sensors and the cost of each HoverBot to be reduced.

Our Linbot uses a voice coil system for actuation, audio output, sensing, and communication. This voice coil system is based on the Wormbot.^16^

### Voice coils for actuation, communication, and sensing

Voice coil systems are electromagnetic systems that are based on the same fundamental principles as loudspeakers; their method of actuation is detailed in Supplementary Figure S1. The voice coil system consists of an actuating electromagnetic coil and a permanent magnet, which are attached together by a soft body that functions as a spring. The electromagnet can repel or attract the permanent magnet and this force stretches or compresses the body of the robot, creating linear motion. The voice coils are hybrid hard/soft systems. Hybrid systems can take advantage of rigid components while still being able to partially conform to their environment.^17^

Voice coils have been shown to be a useful actuation system for soft robotics.^16^ The voice coil systems are multifunctional components that demonstrate different behaviors over a range of different control signal frequencies. In addition to actuation capabilities at low frequencies, the voice coils can function as loudspeakers at audible sound frequencies and can be used for communications at higher frequencies.^16^ This communication is based on inductive data transmission.^18^

We also utilize the permanent magnets in our voice coil system for sensing. The soft body of the robot deforms when it is actuated or when it is subjected to external force and we measure this deformation by monitoring a three-axis Hall-effect sensor, which responds to the relative position of the permanent magnets that are embedded on the top of the robot. This approach allows us to perform both proprioceptive sensing and tactile sensing.^19^

### Building soft modular systems with voice coils

The simple linear action of voice coil systems is similar to the action of myocytes (muscle cells). Myocytes can only perform a simple action, contraction, and respond to simple inputs, signals from a nerve.^20^ Many of these simple pairs of myocytes and nerves, placed at different positions and orientations, can produce complex actions such as prehensile movement or skeletal locomotion. Stacking units in this way can produce useful behavior for robotic applications.^21–24^ In this article, we provide two examples of stacking: first, peristaltic locomotion, which has been a major interest for soft robotic researchers.^25–28^ Second, we rearrange the units used for peristaltic locomotion into a grid to create a peristaltic table that is capable of moving objects over the surface.^29,30^ Two-dimensional (2D) matrices of actuators have been created by Kim *et al*.,^9^ Stanley *et al*.,^10^ and Follmer *et al*.,^11^ and these arrays could be adapted to allow a system designer to make a peristaltic table.

In this article, we have combined both computation and sensing into actuators that we arrange in a 2D array to create a fully distributed and modular peristaltic table.

To allow voice coils to function similarly to nerve/myocyte pairs, they need onboard computation to control their behavior. By including this functionality, we have created linear modular robots that can be stacked together. We have developed low-cost robots—Linbots—that can be configured to produce different forms of useful behavior. Our Linbots are capable of communication, actuation, sensing, and proprioception all through their central voice coil system.

## Linbot Design

### Design of the hardware

**FIG. 1.**
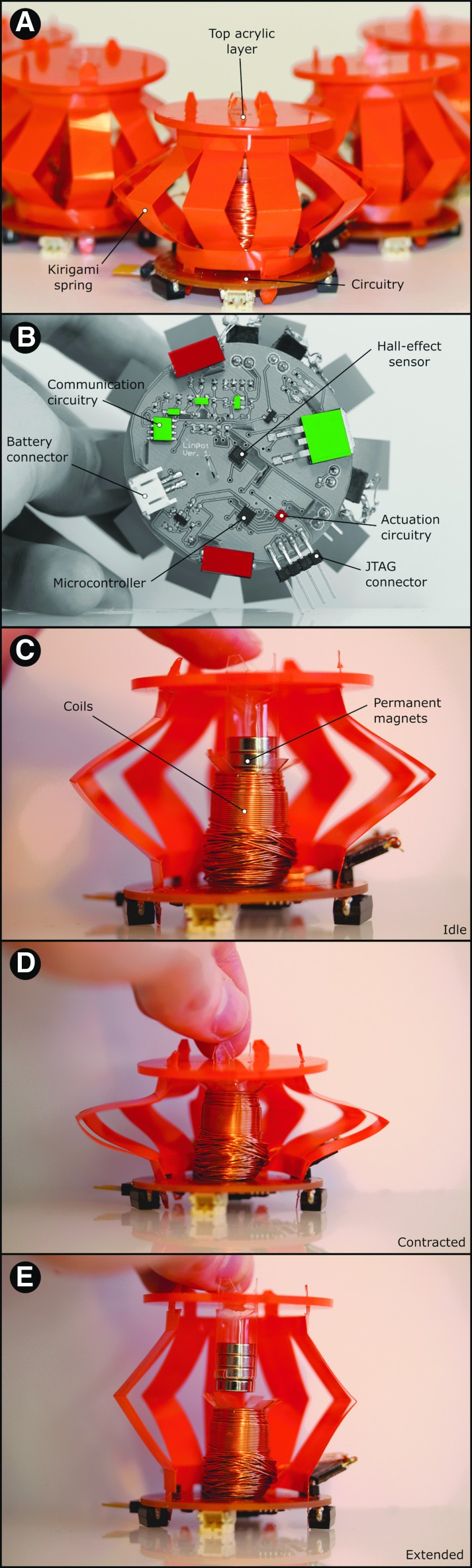
System overview. **(A)** A side view of a Linbot showing the kirigami spring and top acrylic layer. **(B)** A Linbot PCB. **(C)** A cutaway of a Linbot. The coil interacts with the permanent magnets and either pulls or pushes the kirigami spring. The circuitry is embedded on the *bottom side*. **(D)** A Linbot contracting. **(E)** A Linbot extending. PCB, printed circuit board.

Figure 1A shows an individual Linbot unit. The main body of each Linbot is made up of the voice coil system, which includes the following: electromagnetic coils wound around a reel; permanent magnets embedded in a holder; and a spring consisting of connected bent legs resembling a Chinese lantern. A sketch of the Linbot, including its main components, is shown in Supplementary Figure S1. The magnet holder is attached to the spring via a circular, acrylic top layer. The coil reel is attached to the spring via the PCB, which serves as the bottom layer.

The Linbot can be extended or contracted axially from its rest position, depending on the polarity of current applied to the electromagnetic coil in the voice coil system. A sketch of the actuation mechanism is shown in Supplementary Figure S1. The sensing capability of the Linbots is achieved by a combination of a three-axis Hall-effect sensor incorporated in the PCB and the permanent magnets in the voice coil system. The Hall-effect sensor can track the motion of the magnets in three dimensions. This allows the Linbot to function as a tactile sensor since a change in displacement due to the addition or removal of objects on the Linbot can be easily detected.

#### Design of the voice coil system

We made the magnet holder, reel, and spring from acetate sheets using kirigami. Kirigami involves cutting a pattern out of the sheets and folding it into the desired configuration, the 2D patterns are shown in Supplementary Figure S2. We use kirigami as it allows our components to be low cost and highly manufacturable.^31^ The electromagnetic coils consist of two 12-turn coils used for the transmission circuit, and a larger 200-turn coil used for the actuation and receiver circuit. A circuit diagram of the coils is shown in Supplementary Figure S3. Supplementary Figures S4 and S5 show the block diagram and circuit schematic of the transmission and receiver circuits.

We used permanent neodymium magnets in our voice coil system. The internal components of the voice coil system are shown in Figure 1C. Supplementary Figure S6 shows a labeled picture of the custom-built coil-winding machine used for producing the actuation coils of the Linbots.

#### Design of the control electronics

We designed a fully integrated PCB incorporating a transmission circuit, receiver circuit, microcontroller (STM8S), voltage regulator, H-bridge (DRV8837), and Hall-effect sensor (MLX90393) as shown in Figure 1B. The PCB schematic is provided in Supplementary Figure S7. We designed each Linbot to use a single PCB for control, sensing, and communication. The PCB is powered by a 450 mAh 7.4 V lithium polymer battery. Our communication system utilizes electromagnetic induction for data transmission, where the transmission coil generates an alternating magnetic field that induces a voltage in the receiver coil. Further information on the electronic design is detailed in The Electronic Design of the Linbot section of Supplementary Data.

### Design of the control software

We wrote the control software in C and used standard peripheral libraries from ST,^32^ including I^2^C libraries for the Hall-effect sensor and universal asynchronous receiver/transmitter (UART) for communications. The Hall-effect measurements of magnetic field are used to calculate the displacement of the top half of the Linbot from its rest state.

We use the standard UART protocol^33^ for magnetic communication to take advantage of the libraries provided by ST. The UART protocol uses a high idle line, which is pulled low at the start of a message. However, with the Linbot system, if two robots have their transmitters switched in, then all communications will be blocked. Therefore, we need our robots to switch their transmitters off when they are not sending any data. This requirement can lead to an extra byte, with a 0 value, being received at the end of each transmission. To avoid the extra bytes affecting the received data, we check for and remove these erroneous bytes.

## Experimental Design

### Design of the experiments to characterize an individual Linbot

This section discusses the design of the experiments used to demonstrate the capabilities of a single Linbot.

#### Quantifying output force

**FIG. 2.**
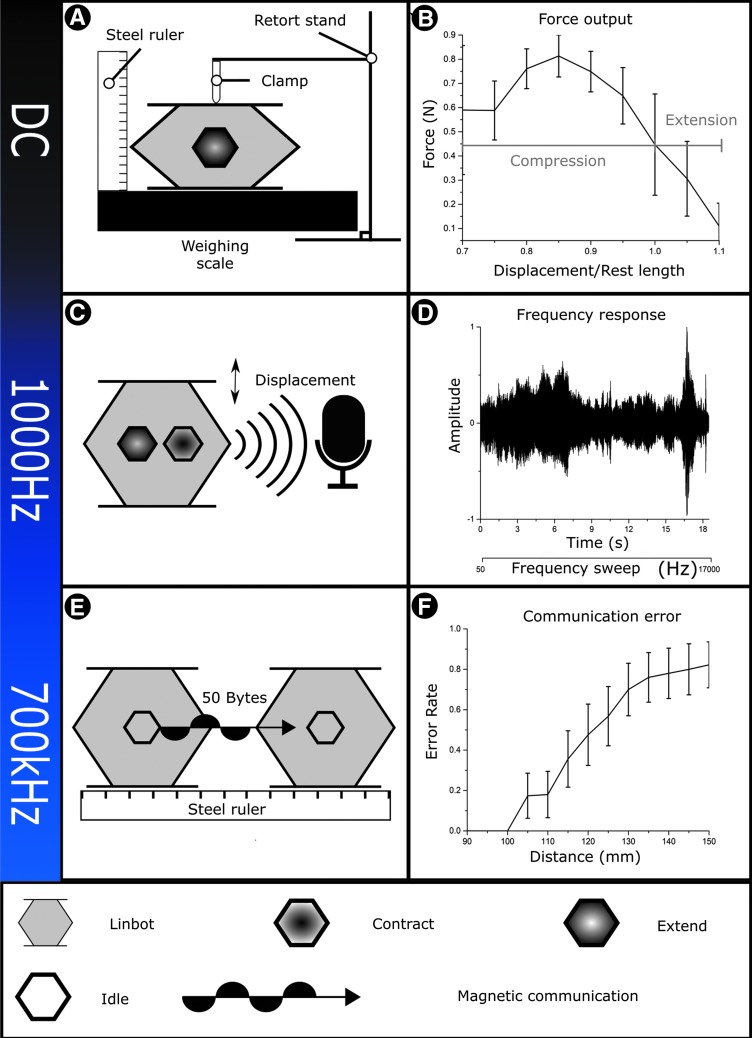
Frequency-dependent functionality. **(A)** A schematic of our experiment to quantify the force output. This experiment involved control frequencies of hertz or lower. **(B)** The force output of the Linbot against relative length. The force output shown in the figure is a combination of the electromagnetic and spring forces of the voice coil. This shows that the Linbot has the highest force output around its rest length. **(C)** A schematic of our frequency response experiment. This experiment involved control frequencies of tens of hertz up to tens of kilohertz. **(D)** The sound wave produced by the Linbot during the last 18 s of a frequency test, shown in Supplementary Video S3. **(E)** A schematic of our experiment to evaluate the communication between Linbots. This experiment involved control frequencies of hundreds of kilohertz. **(F)** The byte error rate. This shows that the Linbots can communicate without error with up to 100 cm between their centers.

To evaluate the output force of the Linbots, we designed a controllable experimental setup with a scale, ruler, and clamp, shown in Figure 2A. Before starting the experiment, we zero the scale with a Linbot, a battery, and a ruler on it. We programmed the Linbot to extend periodically with maximum force. We used a clamp from a retort stand to restrict the height of the Linbot to different relative lengths. Since each of our hand-folded springs has a different rest length, we compare the force to the relative length rather than the absolute length. The relative length is given by the following:

\begin{align*}
{ { { x_r } = \frac { x }  { { { x_0 } } } , }} \tag { 1 } 
\end{align*}

where *x_r_* is the extension, *x* is the absolute length, and *x*_0_ is the rest length. *x*_0_ is measured before starting the experiment. We increase the extension in steps of 0.05 from 0.65 to 1.1; these extensions represent the full range of motion for the Linbots. Using relative length will introduce some error to the experiments as the electromagnetic force from the voice coil will depend on absolute length. The measured mass on the scale was used to calculate the force output of the Linbot at each extension. This output force is a combination of the spring force and electromagnetic force.

We repeated the experiment on a sample of five different Linbots as a screening experiment; this sample size allowed us to measure more than half of the available Linbot population (nine in total). We recorded the average force from these experiments and standard deviation at each extension. One repeat of this experiment is shown in Supplementary Video S2.

#### Frequency response analysis

The primary function of the voice coil system is linear actuation at low frequencies, but can also be used across a wide frequency range to provide audio output or communicate with neighbors. In this experiment, we programmed the microcontroller to vary the coil frequency from 7 Hz to 13.5 kHz. We then recorded sound produced by the Linbot. The experimental setup is depicted in Figure 2C.

To test pulse-width-modulation (PWM) control of the Linbot, we used the microcontroller to provide a PWM signal to the coil. We then observed the effect of changing the duty cycle on the actuation scheme. We first sweep through duty cycles from 0% to 100% and then from 100% to 0%. Next, we step the Linbot between set duty cycles of 10%, 20%, 40%, and 80%. We recorded the experiments using an 18-mp Canon EOS 100D camera and an EF-S 18–55 mm f/3.5–5.6 IS STM lens. We ran this experiment once to demonstrate the capabilities of the Linbots and the output is shown in Supplementary Videos S3 and S4.

#### Feasibility of tactile sensing

We designed an experiment to demonstrate the tactile sensing capabilities of the Linbot by showing that the Hall-effect sensor can be used to track the movement of the magnets in three dimensions. The *Z* axis of the Hall-effect sensor can be used to measure force parallel to the direction of motion of a Linbot. The *X* and *Y* axes of the sensor can measure the shear force between the two halves of the Linbot. In this experiment, the top half of the Linbot is moved along the positive and negative directions of the *X*, *Y*, and *Z* axes. A combination of the different LEDs on the Linbot is used to display the direction of displacement. The schematic of the experiment is shown in Supplementary Figure S8. The experiment was run once to demonstrate the capabilities of the Linbots and is shown in Supplementary Video S5.

### Design of the communication experiment

We designed an experiment to investigate bidirectional communication between two Linbots and to evaluate the error rate in data transmission at different communication distances. The experimental setup is shown in Figure 2E. We demonstrate communication between the Linbots using on–off keying of a 700 kHz carrier signal. The coils were not modified for demonstrating communication in any of the experiments.

In this experiment, we tested the communication between two Linbots at varying distances from one another. We increased the distance between the Linbots in steps of 5 mm from 90 to 150 mm. We measured the distance from the centers of the Linbots. The experiment is divided into two parts, where at each distance, the first Linbot transmits a stream of data and the second Linbot receives the transmitted data, then the second Linbot transmits a stream of data and the first Linbot receives the transmitted data. The transmitted information consists of 50 bytes of data. We set these data to be the numbers 11–211 in incremental steps of 4. This array of numbers varies the shape of the signal.

The Linbot that receives the transmitted data knows the series of bytes it should be receiving, and when it receives a byte it checks the value against its expected value. If the value is different from the expected value, the Linbot interprets the byte as a faulty byte. In the experiment, the orange light depicts a Linbot in transmission mode and a green light depicts a Linbot in receiver mode. After receiving the stream of bytes, if there is a fault in the received data, the Linbot uses a combination of its LEDs to depict the number of faulty bytes received. The Linbot flashes a blue LED once for every 10 faulty bytes and flashes an orange LED once for the remaining single faulty bytes.

The number of wrong bytes received is used to calculate the error rate at each communication distance. If the Linbot does not receive any transmitted data because it is outside the communication range, the error rate is considered to be 1. Once a distance is reached where both Linbots have an error rate of 1, all larger distances for that pair are assumed to be 1.

Due to the large number of Linbots, we decided to perform a screening experiment by sampling more than 50% of the population, so that we could gain a good understanding of the overall capability of the Linbots. We chose five Linbots at random, from the population of nine, and used each one for two tests at each range. To separate the effects of transmission strength from the receiving sensitivity for each Linbot, we chose to test each Linbot twice, in a different pairing. By taking into account all of the permutations we calculated that we needed 500 binary tests of communication at each range. This experiment is shown in Supplementary Video S6.

### Design of the Linbot collective experiments

#### Design of the peristaltic conveyor

**FIG. 3.**
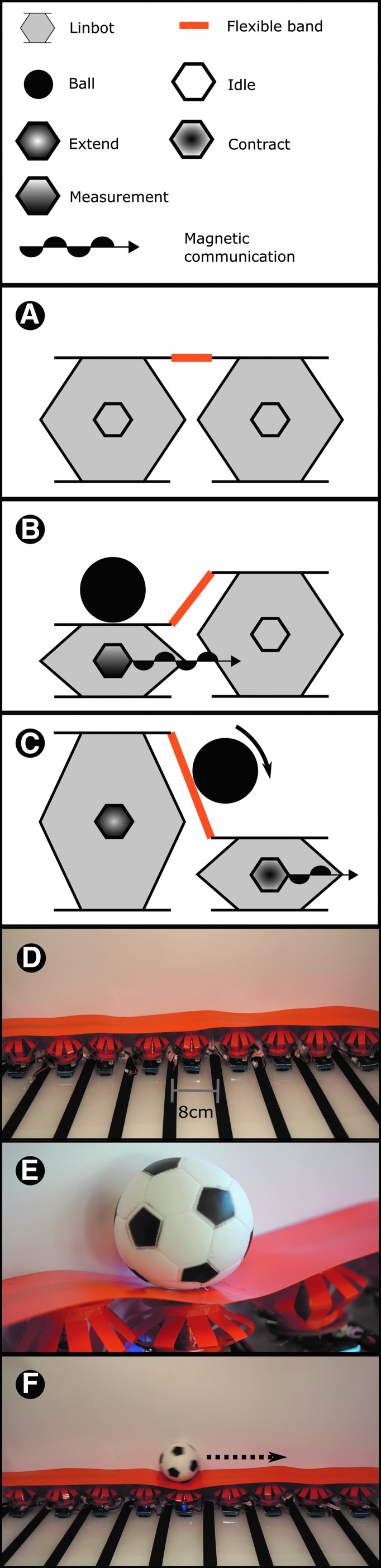
Peristaltic conveyor. **(A)** Schematic of two Linbots at rest within the peristaltic conveyor. **(B)** Schematic of the same Linbots within the conveyor detecting a weight placed on the first Linbot. At this point, it communicates with its neighbor. **(C)** Schematic of the two Linbots after the first Linbot sends the communication signal. This communication causes the second Linbot to contract, while the first Linbot extends. The actuation changes the slope of the flexible band and causes the weight to roll off and onto the second Linbot. The second Linbot then communicates to its next neighbor along the conveyor. This process is repeated to create a traveling wave along the conveyor. **(D)** The conveyor at rest. **(E)** A close-up of a ball traveling along the conveyor. **(F)** The conveyor moving a ball from one side to the other.

In addition to the experiments that show the capabilities of one or two Linbots, we performed additional demonstrations to show how the Linbots can perform collective behaviors. For the first demonstration, we designed a peristaltic conveyor to show how a collective of our Linbots can use simple individual behaviors to perform a more complex task. The system is shown in Figure 3D. The conveyor used nine Linbots arranged in a straight line within a platform. The current used to power the Linbot communication controls the maximum range of the communication. Based on the range of around 100 mm seen in the communication test, we placed the Linbots 80 mm apart. This spacing only allowed nearest neighbor communication.

The platform consists of two parts: a backboard to prevent objects from falling off the conveyor and a base to hold the Linbots. The conveyor was given a 4° roll so that conveyed objects rest against the backboard, and a 1° pitch, so that these objects are moved uphill. For this experiment, we programmed the Linbot as a finite state machine to respond to either contact by an object or receiving a message from a neighbor. On detecting communication from another Linbot, the receiving Linbot contracts. When the Linbot contracts or is compressed, it turns on its communication and transmits information to its neighboring Linbot. The Linbot then expands to push the object to the next Linbot. Multiple Linbots performing this sequence create a traveling wave along the conveyor from one side to the other.

After expansion, each Linbot enters an unresponsive state for a set amount of time to avoid responding to the signal from the succeeding Linbot in the conveyor. This mechanism ensures that the wave travels only in one direction, carrying the object with it. Schematics of the mechanism are shown in Figure 3A–C. We used a small toy ball that weighs 40 g for this experiment. This experiment is shown in Supplementary Video S7.

#### Design of the peristaltic sorter

**FIG. 4.**
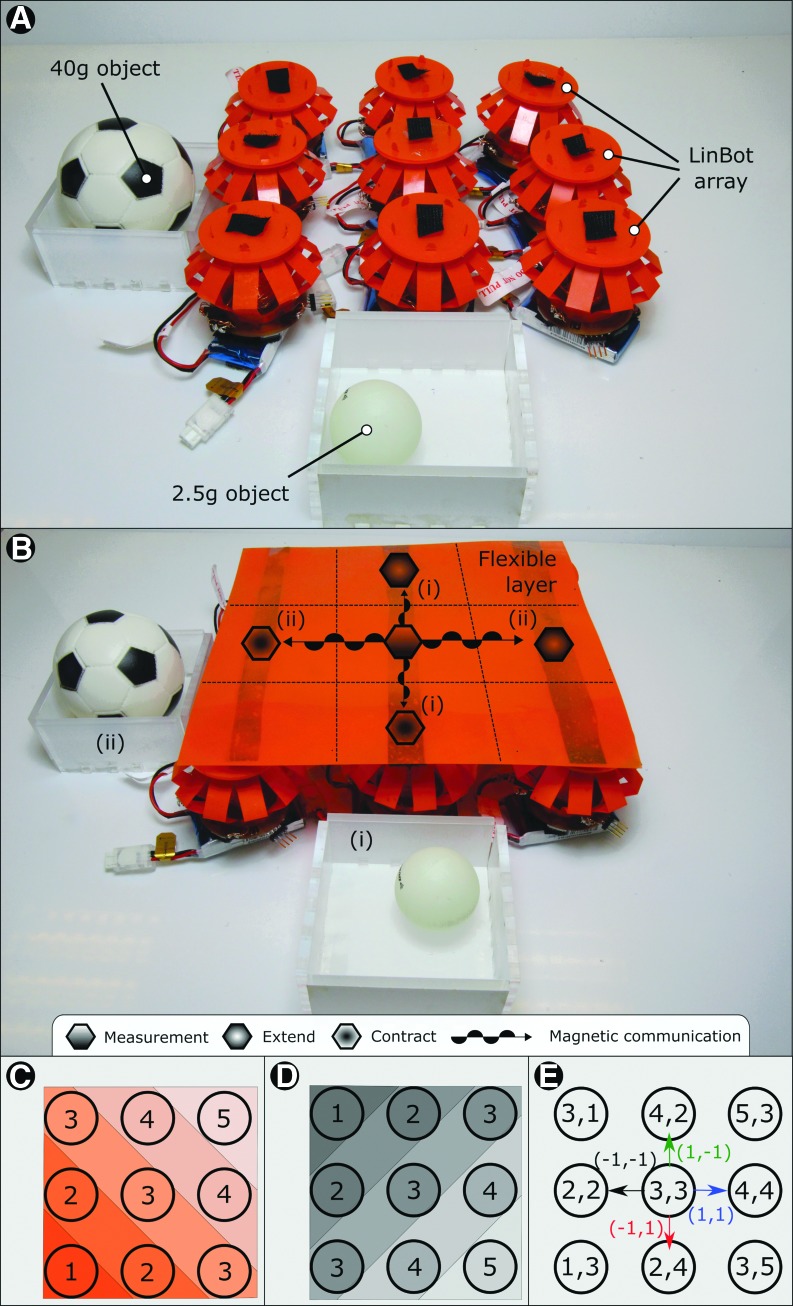
Peristaltic sorter and addressing system. **(A)** The peristaltic sorter without the flexible layer on *top* of it, showing the Linbot array. **(B)** The peristaltic sorter with the flexible layer attached. The behavior of the sorter is shown with the central Linbot detecting the weight of an object and its neighbors actuating to roll the object in the desired direction based on weight. (i) Behavior of the sorter for the 2.5 g object. (ii) Behavior of the sorter for the 40 g object. All the Linbots in the sorting system had the same controller. **(C)** Shows the allocation of the first coordinate of the addressing system, based on communication hops from the *bottom left* Linbot. **(D)** Shows the allocation of the second coordinate of the addressing system, based on communication hops from the *top left* Linbot. **(E)** The final addresses for the peristaltic sorter. Vectors between neighboring Linbots in this coordinate scheme are shown around the central Linbot.

To demonstrate how Linbots can be reconfigured to perform other tasks, we designed a peristaltic sorter using the Linbots. The layout of the Linbots for this sorter is shown in Figure 4A. In this demonstration, nine Linbots are arranged in a 3 × 3 array and are capable of sorting objects by their weight. A 2.5 g ball and a 40 g ball were used to demonstrate the sorting technique in this experiment. The 2.5 g ball should be moved to the left, and the 40 g ball should be moved downward.

As the sorter configuration uses multiple, nondormant Linbots within the communication range of one another, we needed to use an addressing system to control if a received command activates a Linbot. To implement the addressing system before starting the sorting task, when the Linbot collective is turned on, the bottom left Linbot is pressed. Pressing a Linbot will cause it to propagate a message through the collective, allowing each Linbot to know how far away, on the grid, it is from the Linbot pressed. The process is repeated with the top left Linbot. Based on the grid distances given by the bottom left and top left Linbot, each Linbot will have unique coordinates. Each Linbot will also know the relative position of the other coordinates on the grid. This addressing system is demonstrated in Figure 4C–E.

When a Linbot detects a weight placed on it, it increments a counter related to the class of the weight it believes is on top of it. When the counter of a class reaches a threshold, the Linbot sends a message to its neighbors causing them to actuate. This buildup and discharge behavior is inspired by action potentials in heart muscle cells.^34^

The message transmitted by the Linbot under a weight contains the coordinates of the signaling Linbot and the direction to move the weight. The neighboring Linbots actuate based on their relative position to the instructing Linbot. This actuation leads to the ball rolling off the edge of the grid in the desired direction. The Linbots all have the same controller and can function in any role or position within the system. The setup and behavior of the sorter are depicted in Figure 4B. This behavior is similar to the peristaltic conveyor, detailed in the Design of the Communication Experiment section, and rolls the ball in the correct direction.

## Results and Discussion

### Fabrication and assembly

We designed the Linbots to have a size and weight appropriate for ease of fabrication, manufacturing, and assembly. They each have an inner diameter of 50 mm, an outer diameter of 70 mm, and a height of 50 mm at the rest position. This size allowed us to fit all of the electronic components on one side of the PCB while also allowing large numbers of Linbots to be space efficient. Each Linbot weighs 33 g, and the battery used for each Linbot also weighs 33 g. The Linbots are designed for manufacturability and the total bill of materials is £13.64. Further information about the components and assembly can be found in The Fabrication of the Linbot section of Supplementary Data, and in the supplementary ZIP folder containing CAD files and PCB schematics.

### Results of the individual Linbot characterization experiments

#### Quantifying force output

We expected the force output to be the sum of spring force and magnetic force between the electromagnets and permanent magnets. When the coil and magnet are fully separated, the force is given by the following:

\begin{align*}
{ { F \simeq kd \,+ \; \mu { \frac { { q_ { coil } } { q_ { pmag } } }  { 4 \pi { { \left( { d + { r_0 } } \right) } ^2 } } } , }} \tag { 2 } 
\end{align*}

where *F* is the total force, *k* is the spring constant, *d* is the distance from rest position, *μ* is the permittivity of air, ${q_{coil}}$ is the pole strength of the electromagnetic coil, ${q_{pmag}}$ is the pole strength of the permanent magnets, and *r*_0_ is the initial distance between center of the coil and center of the magnets. *q_coil_* is given by the following:

\begin{align*}
{ { { q_ { coil } } = \frac { { NIA } }  { L } \; , }} \tag { 3 } 
\end{align*}

where *N* is the number of turns, *I* is the current through the coil, *A* is the cross-sectional area of the coil, and *L* is the length of the coil.

When the permanent magnets enter the coil, the magnetic force between them drops, reaching zero when their centers align. At the beginning, we expect a positive force trending down linearly due to the spring force dominating. Once the centers of the permanent magnets move away from the center of the coil, we expect a rapid increase that overtakes the decrease of the spring force. Once the permanent magnets are separated from the electromagnet, further behavior will be an inverse-square relationship, summed with a negative linear term as the spring force starts pulling the magnet back.

We see this expected behavior in the results from our force test, shown in Figure 2B. One repeat of the experiment is recorded in Supplementary Video S2. The results have a standard deviation of between 0.05 and 0.21 N, and this variance is likely caused by differences during construction, leading to a spread of restoring forces in the springs for each Linbot. The springs can also exhibit some asymmetry, diverting the force from being vertically upward, leading to a reduction in measured force output.

The rest length of the Linbots used in the experiment ranged 46.5–51.0 ± 0.5 mm. Removing this variance would require using a machine to fold the springs of each Linbot. The error seen in this experiment is acceptable as these robots are soft and have not been designed to require a high level of precision to achieve their tasks; instead, they rely on compliance to adapt to the environment. The maximum force output was 0.81 N, which suggests that the Linbots could lift objects with masses below ∼83 g. For objects near this limit, lifting would require a controller that actuates such that it does not allow the Linbot to compress below 0.85 relative length. Below this relative length, the force output drops off and the Linbot may be unable to lift as heavy an object.

#### Frequency response analysis

We expected the Linbots to actuate at a low control frequency and transition to sound output as the frequency reaches audible levels. At high frequency, we also expect to use PWM to produce forces smaller than the maximum actuation force, and therefore, partial actuation. We demonstrated that the Linbots can actuate up to high frequencies and act as a loudspeaker. The waveform for the output sound is shown in Figure 2D, and its frequency spectrum is shown in Supplementary Figure S9. We also show this behavior in Supplementary Video S3. The video shows the Linbot actuating at increasing frequencies and then transitioning to sound output at the highest frequencies. Our use of a PWM actuation signal produced the expected partial actuation behavior. The partial actuation behavior resulting from the PWM signal is demonstrated in Supplementary Video S4.

#### Feasibility of tactile sensing

We designed the Linbots to be able to sense the displacement of their top half relative to their bottom half. We expected accurate classification of movement for positive and negative movements along three orthogonal axes. We demonstrated this ability in Supplementary Video S5. The video shows the Linbot being moved in six orthogonal directions, the direction of movement is displayed via the LED color combinations, shown in Supplementary Figure S8.

### Evaluation of the communication between Linbots

We expected each pair of Linbots to show a very high successful transmission rate at separation distances below their maximum transmission range, as the receiving circuits have a threshold for signal strength that is set above the background noise. The background noise level from the receiver circuit is shown by Supplementary Figure S12. As they reach their maximum communication range, we expected their success rate to decrease sharply. This decrease is due to the received transmission strength approaching the threshold of the receiver, which is detailed in The Fabrication of the Linbot section of Supplementary Data. As some signals are pushed over or under the threshold by random noise, we would expect many faulty bytes. Once above the maximum transmission range, we expect none of the bytes to be received, as the transmission strength would always be below the signal threshold.

Due to variation in the construction of the coils, the transmission strength and receiving sensitivity vary in each Linbot. This variation gives each pair of Linbots a different maximum transmission range. The effect of this variation was expected to give rise to a high success region before any pairs reach their maximum range, then a distance where the average success rate is below 1 and has a high error due to some pairs having started their rapid decrease, while others have not. The average success rate should then decrease to zero as more pairs move past their maximum range.

The results of the communication test between the Linbots are shown in Figure 2F. The experiment is shown in Supplementary Video S6. We demonstrated that the Linbots can communicate with each other over at least 100 cm, giving us our expected high success region. Beyond that, the average success rate started to decline with a high error rate as expected. The shortest maximum transmission range seen was 105 mm, while the longest was over 150 mm. One Linbot did not reach a maximum transmission range. This unexpectedly high maximum range is the reason the mean error rate only reaches ∼0.8.

The results of these communication experiments are used to quantify the maximum distance for reliable communications and acted as a guideline for the separation distance between Linbots in the following experiments that used collectives of robots.

### Results of the Linbot collective experiments

#### Peristaltic conveyor

We expected a collective of Linbots to be able to generate a sufficiently powerful peristaltic wave to roll a ball up a gradient. Our Linbot collective achieved this task and the result can be seen in Supplementary Videos S7 and S8. These videos show the Linbots rolling a ball along the conveyor by using collective behavior. We used a delay of 0.45 s before each Linbot propagated the wave, and this delay led to the ball traveling at an average of 18 cm/s across the conveyor. A close-up of the ball moving along the conveyor and the conveyor moving the ball from one side to the other is shown in Figure 3E and F, respectively.

This conveyor would be suitable for any objects that slide or roll but would have trouble moving adhesive objects. The peristaltic wave used could have been faster, as the video shows the ball sometimes getting slowed by falling into the trough of the wave. A faster wave also increases the chance that the ball would fall behind the wave and stop. The speed of the wave is limited by the gradient created for the ball to roll down. This gradient is determined by the actuation range of the Linbots and the spacing between them. In this experiment, the angle of the rising slope of the peristaltic wave is ∼12°, including the opposing 3° pitch of the conveyor. Increasing the range of motion of the Linbots or reducing the distance between them would allow for a faster peristaltic wave.

#### Two-dimensional peristaltic sorting table

We designed the Linbot collectives to be able to sense the difference between two balls, one weighing 2.5 g and the other 40 g. We also expected that they would move the 2.5 g balls off the grid to the left side and the 40 g ball off the grid to the bottom side. Our collective behaved as expected. This behavior is depicted in Figure 4B and can be seen in Supplementary Videos S9 and S10. The videos show the Linbots rolling the two balls in the correct directions.

The signal used to classify the balls is shown in Supplementary Figures S10 and S11. The difference in signal between the two balls means that comparing the signal to a threshold spaced between the two will correctly classify a ball every time. These figures also show that the rest length of a Linbot is changed even after removing the weight placed on them. In the experiment, we removed the effect of this change on the signal by resetting the base Hall-effect reading after a weight is removed. The Linbots in this sorter have identical controllers that use the relative positions of Linbots to determine actions. This distributed control means that the controller is not reliant on the size of the sorter.

## Scope for Development

### Vibrational sorting

The high actuation frequency of the Linbots allows them to be used for vibrational sorting. Vibrating collections of objects allow them to be sorted by firmness or density. A single Linbot with a slanted plate attached to the top can produce the vibrations needed to sort objects. The peristaltic sorter could also be adapted for a mixture of vibrational and peristaltic sorting.

### Communication range

Our use of inductive data transmission for the Linbots means that communication is only possible between nearby Linbots. In the future, the frequency of the carrier wave used could be increased to create far-field communication between Linbots and to enable long-range communication between dispersed Linbots.

### Wireless charging

Through adding a rectifier and LiPo balance circuit to the Linbot, its battery could be charged through inductive power transfer. This would involve placing the Linbot in a strong alternating magnetic field, whose power would be coupled into the main coil of the Linbot.

### Wireless programming

By writing a program to allow Linbots to be programmed via UART, we could allow for wireless programming of the Linbots. This modification would reduce the time needed to program the Linbots as an antenna could be used to program them simultaneously. It would also allow new programs and controllers to be propagated through a collective by the Linbots themselves.

### Additional sensors

By connecting I^2^C sensors to Linbot PCB I^2^C bus pins, we can tailor the Linbots to new tasks. For example, to make an array of Linbots respond to the distance from objects, IR range sensors can be added to a Linbot PCB.

### Learned behavior

In large Linbot collectives, each individual robot can affect the state of other Linbots with whom it does not have a direct communication link. This complication leads to difficulties in writing effective controllers. Machine learning algorithms could be applied to allow Linbots to learn the dynamics of their collective and even to have individually tailored controllers.

### Self-synchronization

If we have a task that requires synchronization, we can design our Linbot controllers to create a global clock. Using phase rate equalization, any detectable pulses can be used to synchronize the Linbots.^35^ We can create these pulses with periodic broadcasts from a Linbot to neighboring Linbots. We can also use the periodic actuation of a Linbot, which can detect the actuation of neighboring Linbots through tactile signals from a flexible layer on top of them.

### A larger peristaltic sorter

Further robots could be added to make the peristaltic sorter larger without needing to rewrite the controller. This larger sorter could be used to examine large-scale behaviors of Linbots.

### Locomoting Linbot collectives

A Linbot collective could be reconfigured for locomotion, similar to the Wormbot.^16^ The peristaltic conveyor could also be inverted to produce waves that would enable the whole assembly to locomote across a surface. Reducing the weight of the Linbots by decreasing the battery size would allow for more robust locomotion. A 2D Linbot collective designed for locomotion would be capable of steering in response to sensory information.

### Miniaturized Linbots

The Linbots could be miniaturized for use in different applications such as tactile displays or implantable devices. By converting the circuitry into one integrated circuit, the volume of a Linbot could be reduced to about a cubic centimeter. At this size, the Linbots could use the magnets and coils from the smallest commercially available solenoids, while still using off-the-shelf batteries.

Making the robot even smaller would preclude the use of available batteries. This means that either a miniature battery would need to be manufactured or the robot would need to run on miniature charged capacitors and an external power source. At smaller scales, the coil would need to be changed from being a wound wire to a planar coil cut into a PCB, and the magnets would need to be fabricated rather than bought. The limit at this level would be the minimum size of integrated circuits, which means the Linbot cannot be smaller than a few millimeters in size.

## Conclusions

Modular robotic collectives are more robust than monolithic systems. Often modular systems are limited in their functionality due to cost. Soft, modular robots also often require an outside pressure or vacuum source. We present our Linbots, our combination of untethered, reconfigurable robots with soft robotics. Our Linbots demonstrate communication, actuation, tactile sensing, proprioception, and sound synthesis. The linear motion of our Linbots sets them apart from existing modular robots and allows them to be used for tasks that require peristaltic motion.

We demonstrated the abilities of individual Linbots as well as their ability to communicate with one another. We used a Linbot collective to convey a ball up a slope using a peristaltic wave. We then reconfigured this Linbot collective to demonstrate sorting. The collective was able to sort two balls based on weight and transfer them to desired bins.

Our use of multifunctional hardware allows the system to be low cost without sacrificing functionality. The soft nature of our Linbots allows them to conform to their surroundings. Our Linbots provide a low-cost modular platform that can be configured for different real-world tasks. In addition, our multifunctional voice coil system can be adapted to other modular or swarm robotic systems. Our work supports the move toward reconfigurable, modular robotic platforms as useful tools for both academia and industry.

## Funding

This study was supported by EPSRC via the Robotarium Capital Equipment and CDT Capital Equipment Grants (EP/L016834/1), and the CDT in Robotics and Autonomous Systems. M.P.N. gratefully acknowledges support from the CDT in Intelligent Sensing and Measurement (EP/L016753/1), United Kingdom. A.A.S. acknowledges support from the EPSRC ORCA Hub (EP/R026173/1).

## Supplementary Material

Supplemental data

Supplemental data

Supplemental data

Supplemental data

Supplemental data

Supplemental data

Supplemental data

Supplemental data

Supplemental data

Supplemental data

Supplemental data
