# Supplementary material for: Linbots: Soft Modular Robots Utilizing Voice Coils
Source: Soft Robot. 2019 Apr 16;6(2):195–205. doi: 10.1089/soro.2018.0058 (PMC6486669; doi:10.1089/soro.2018.0058)
Supplement: Supplemental data [file Supp_Data.zip › Supp_Data.pdf]

Supplementary Data

The Electronic Design of the Linbot

Our communication system contains an inductor-capacitor oscillator, which sustains oscillations at the resonant frequency of the circuit and allows us to produce a carrier wave from a direct current source. Our oscillator uses two 12-turn coils; one coil is used in a resonant circuit to produce an oscillating magnetic field for communication and the other coil, a trigger coil, is coupled to this field and controls the gain of a bipolar junction transistor that drives the resonant circuit. We demonstrated successful communication between Linbots using on–off keying of a 700 kHz carrier signal and a baud rate of $1000\,s^{-1}$. The transmission circuit uses an MOSFET transistor (which is driven by the microcontroller) as a switch to turn the transmission on and off. The transmission signal is stepped up into a higher voltage by the larger 200-turn coil.

The oscillating field induces a signal in the millivolt range in the coils of nearby Linbots. We amplify the signal by passing it through two cascaded class-A amplifiers. We then pass the signal through an envelope detector and then feed it to a comparator. We set the comparator to use a threshold of 16 mV to create a square wave matching the transmitted data. The peaks of the noise generated by the receiving circuit can reach $\sim$14 mV, meaning that the comparator will only pull high on receiving a real incoming transmission, an example waveform of the noise is shown in Supplementary Figure S12. We pass this square wave signal to the microcontroller, where we can recreate the original message.

In addition to receiving the transmitted data, we use the 200-turn coil for actuation. Application of current to the coil allows the Linbot to be contracted or extended along its central axis from its rest position; the direction of actuation depends on the polarity of the current applied to the coil. The bidirectional actuation is shown in Figure 1D and E. The applied current induces a magnetic field in the coil, which either attracts or repels the permanent magnets resulting in this actuation mechanism. We use the microcontroller to control both the frequency of the actuation and the direction of the actuation via the H-bridge driver.

The Hall-effect sensor is controlled by the microcontroller through an I^2C bus. There is an additional pair of header pins included on the Linbot printed circuit board (PCB). These header pins are connected to the microcontroller I^2C peripheral and allow for extra sensors to be easily added to the Linbot.

We incorporated a programming port into the Linbot PCB. We program the Linbot via an ST-Link/V2 debugger using a single wire interface module interface.

The Fabrication of the Linbot

Cost and functionality were the most important factors considered when designing the Linbots. Our rationale behind the system design was to keep the costs as low as possible without sacrificing functionality. The cost of a single Linbot PCB is £7.70 in a batch of 10. The cost of the electronic components associated with a Linbot PCB is £5.94 in a batch of 10.

We purchased the 0.35 mm insulated copper wire and 10 mm permanent neodymium magnets from RS Components. Each Linbot requires three coils: one 200-turn coil and two 12-turn coils. We use our custom-made coil-winding machine, shown in Supplementary Figure S5, to wind these coils. The resulting structure has an inner diameter of 14.5 mm, an outer diameter of 18.5 mm, and a height of 22 mm. Our machine feeds the wire onto a rotating coil holder and we deposit superglue on the wire as it runs so that the coil holds its shape.

We fabricated the top layer of the Linbot from a 3 mm acrylic sheet. We cut the patterns for the kirigami components and top layer using a laser cutter (Epilog Laser Fusion 32). We designed the Linbot PCBs using Eagle PCB Design Software and fabricate them on double-sided Cu-FR4-Cu 0.1-mm boards using an external company called Ragworm (Kent, United Kingdom). We purchased the Hall-effect sensors, MLX90393 (Micropower Triaxis Magnetometer), from Mouser Electronics and soldered them onto the Linbot PCBs to provide sensing abilities.

The Linbots have a minimum battery life of 22.5 min and a maximum of 280 h. The minimum battery life is calculated by assuming constant actuation, which uses a current of 1.2 A. The maximum battery life is calculated by assuming the Linbot is in sleep mode, where it consumes an average current of 1.6 mA. In this mode, the battery life of a Linbot is more than 11 days. The other Linbot capabilities have different current consumption levels and thus can change the battery life. Communication with other Linbots draws 80 mA. The current consumed by the Linbot when functioning as a speaker at maximum volume is 1.2 A. Using the Linbot for tactile sensing will use a current of 8.7 mA. Therefore, the battery life of a Linbot running any of these functionalities or running several functionalities together can be easily calculated. In this Linbot version, we detach the lithium polymer batteries for charging. We charge the batteries using a Linkman lithium battery charger and a 2S–6S balanced charging plate.

Technical Files

The authors have provided a zip-file for CAD files and PCB schematics.

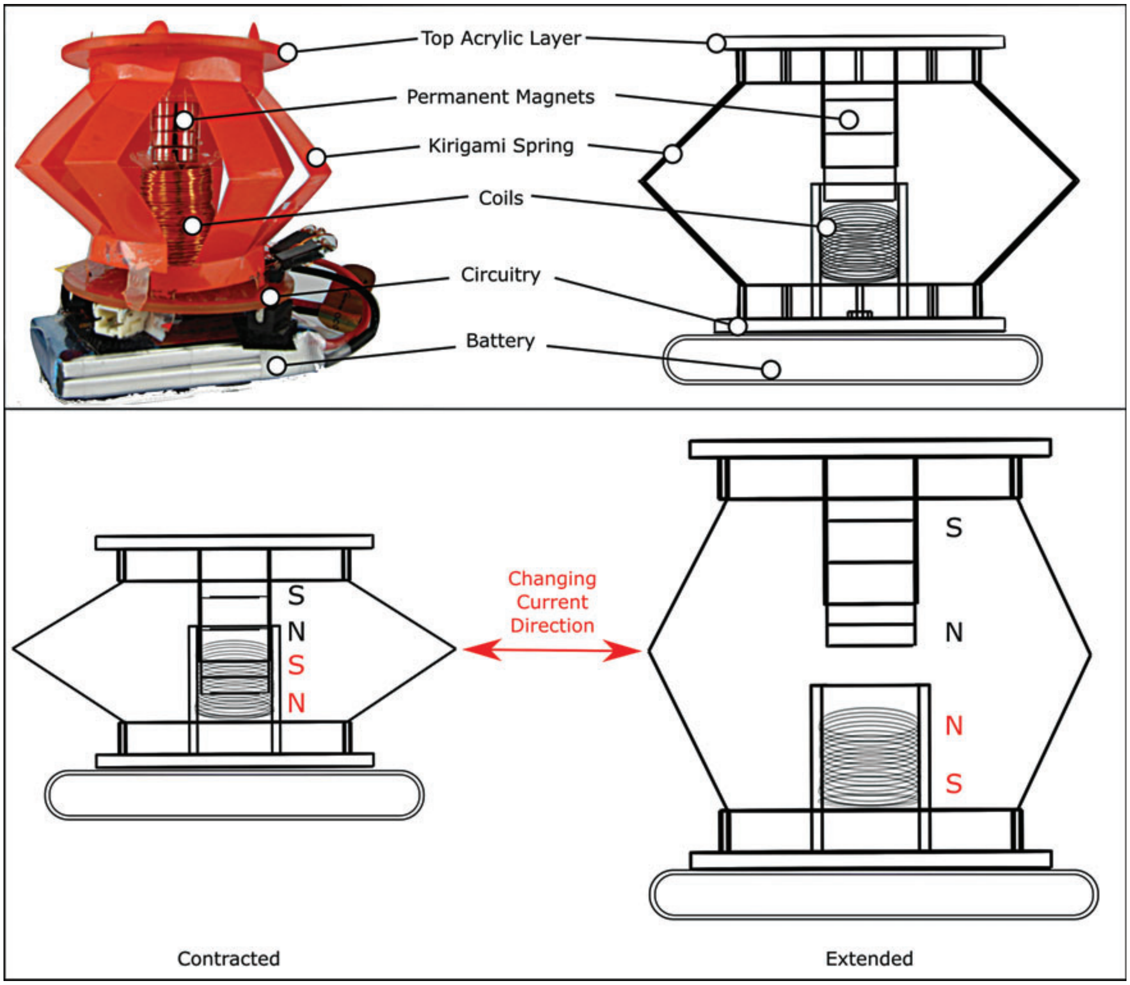

SUPPLEMENTARY FIG. S1. A labeled picture and sketch of the Linbot showing all of its components and a sketch of the actuation mechanism.

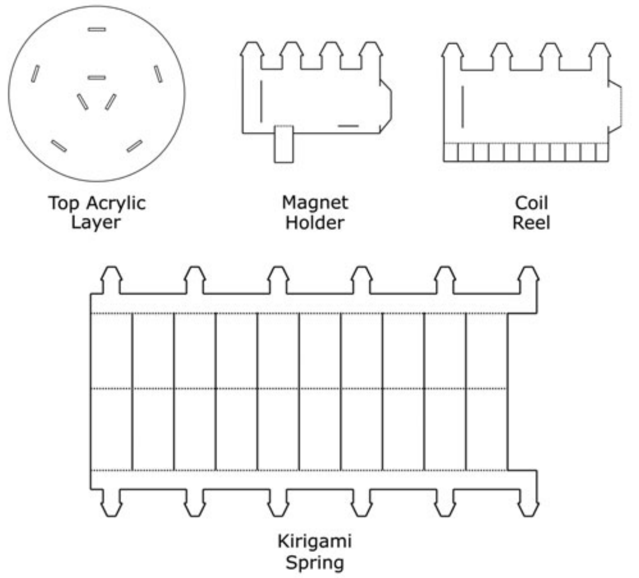

SUPPLEMENTARY FIG. S2. Two-dimensional design of the top acrylic layer, magnet holder, coil reel, and Kirigami spring.

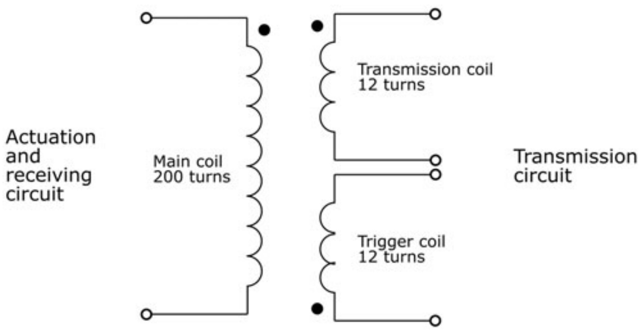

SUPPLEMENTARY FIG. S3. A circuit diagram of the transmission, actuation, and receiving coils.

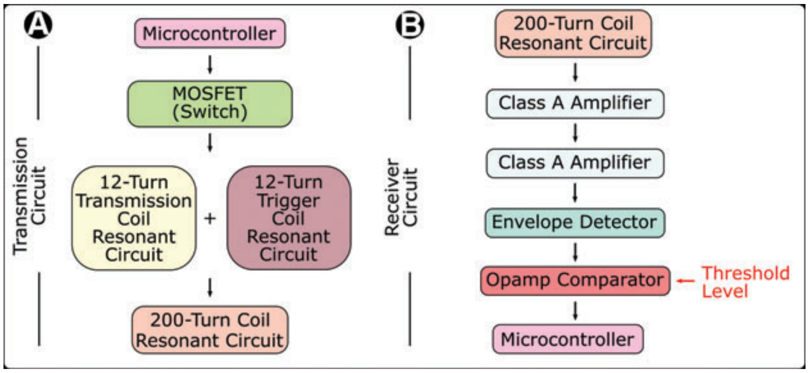

SUPPLEMENTARY FIG. S4. A block diagram of the **(A)** transmission and **(B)** receiver circuits.

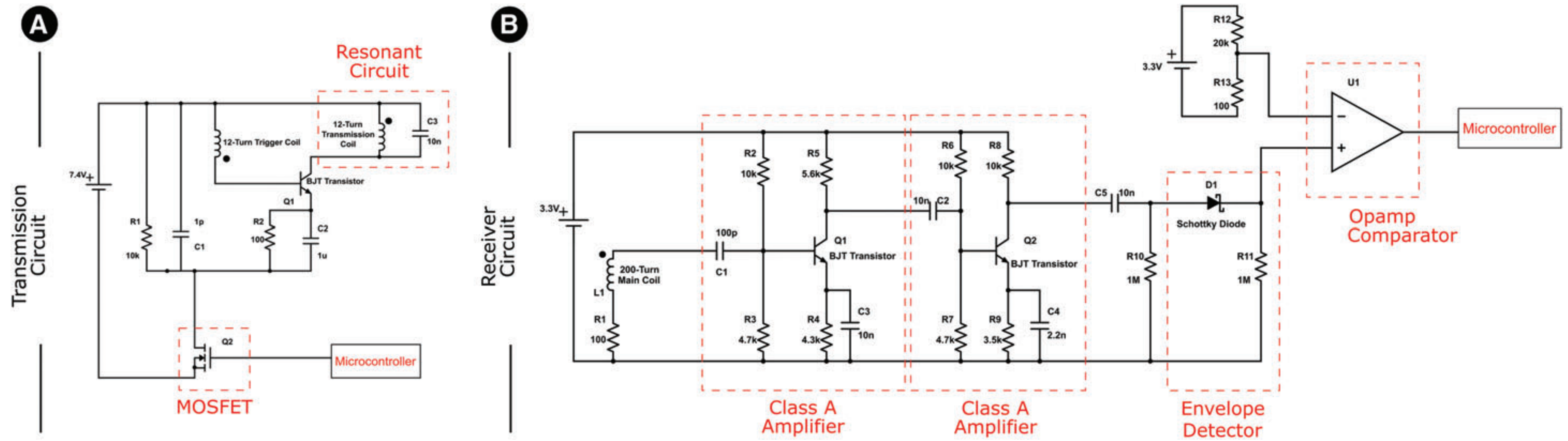

SUPPLEMENTARY FIG. S5. **(A)** Circuit schematic of the transmission circuit. The transmission circuit uses an MOSFET transistor (which is driven by the microcontroller) as a switch to turn the transmission on and off. The transmission signal is stepped up into a higher voltage by the larger 200-turn coil. The coupling between the coils can be found in Supplementary Figure S3. **(B)** Circuit schematic of the receiver circuit. The 200-turn coil is used for receiving the transmitted signal. We amplify the signal by passing it through two cascaded class-A amplifiers. We then pass the signal through an envelope detector and then feed it to a comparator. We set the comparator to use a threshold of 16 mV to create a square wave matching the transmitted data.

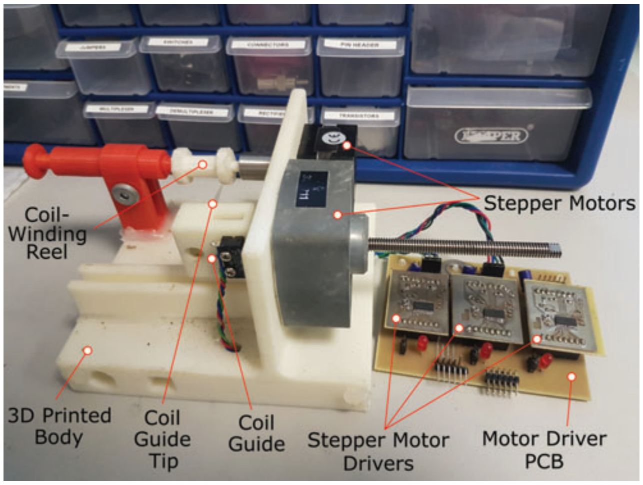

SUPPLEMENTARY FIG. S6. A labeled picture of the custom-built coil-winding machine used for producing the actuation coils of the Linbots.

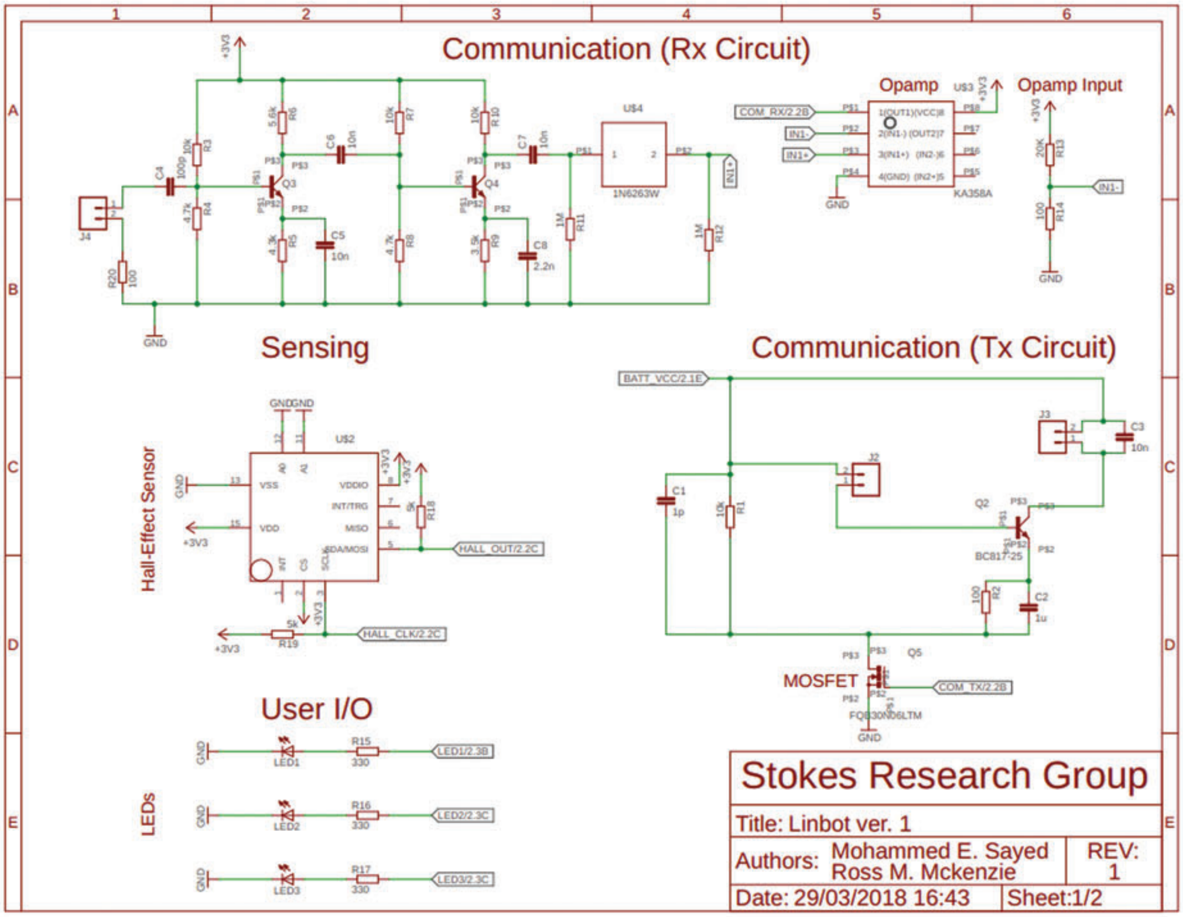

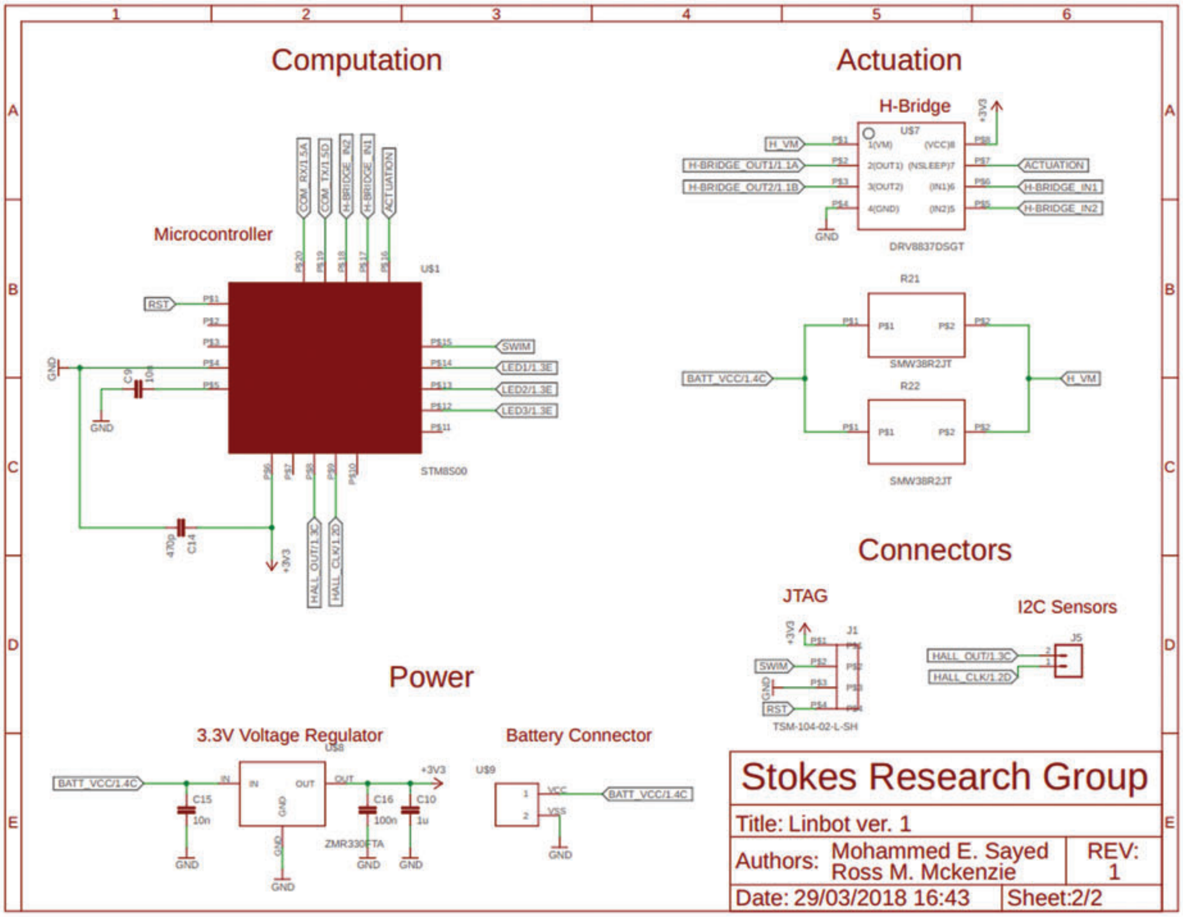

SUPPLEMENTARY FIG. S7. PCB schematic of a Linbot. PCB, printed circuit board.

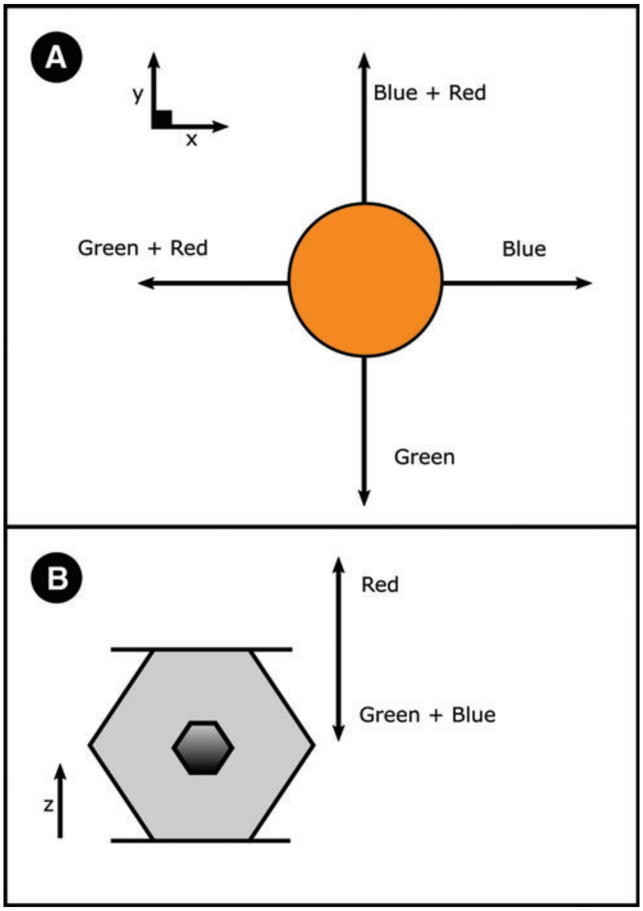

SUPPLEMENTARY FIG. S8. Tactile sensing experiment schematic showing the LED combinations displayed when the Linbot detects displacement **(A)** horizontally and **(B)** vertically.

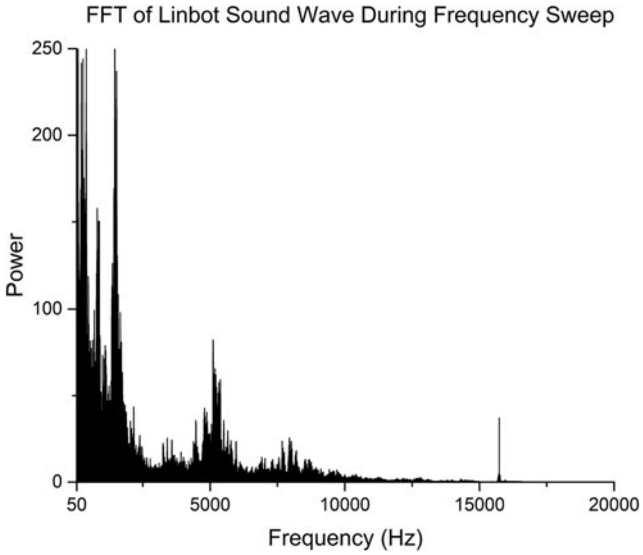

SUPPLEMENTARY FIG. S9. FFT of the sound wave produced by the Linbot during the frequency response experiment. The experiment is shown in Supplementary Video S3. FFT, fast Fourier transform.

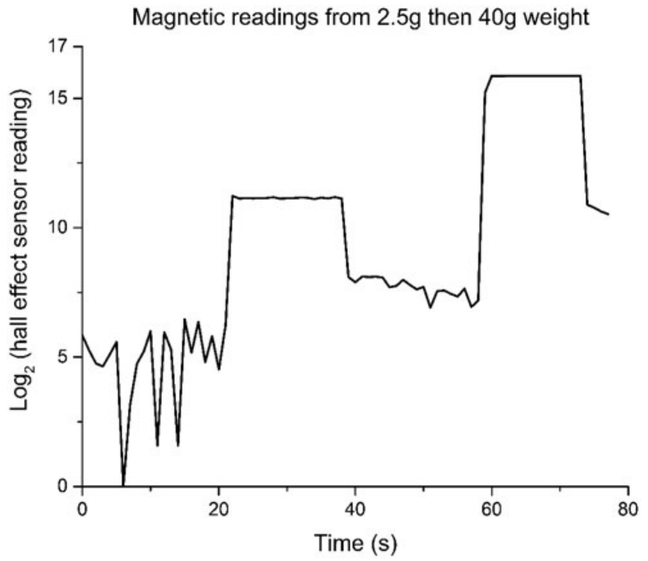

SUPPLEMENTARY FIG. S10. A base 2 logarithmic plot of Hall-effect signal. The signal between 22 and 38 s represents a 2.5 g weight placed on a Linbot. The signal between 59 and 73 s represents a 40 g weight placed on the Linbot. The sampling rate was limited to 1 Hz by the connection between the Linbot and a computer, while the true sampling rate of the Hall-effect sensor is in the hundreds of hertz. The signal representing the Linbot with no weight on it changes after placing a weight on the Linbot.

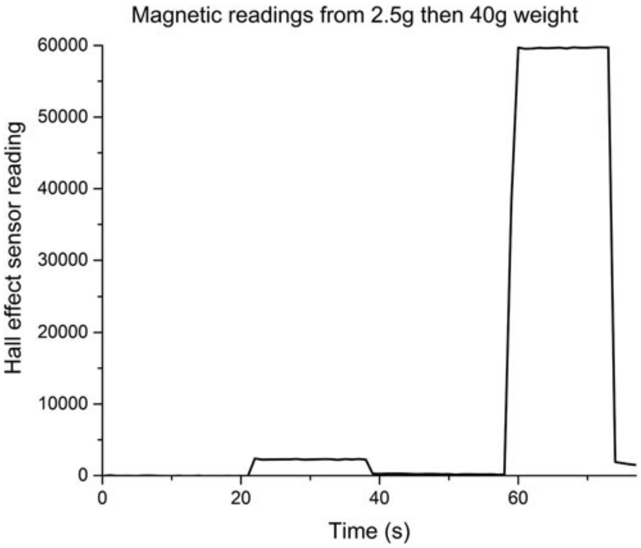

SUPPLEMENTARY FIG. S11. A linear plot of Hall-effect signal. The signal between 22 and 38 s represents a 2.5 g weight placed on a Linbot. The signal between 59 and 73 s represents a 40 g weight placed on the Linbot. The sampling rate was limited to 1 Hz by the connection between the Linbot and a computer, while the true sampling rate of the Hall-effect sensor is in the hundreds of hertz. The signal representing the Linbot with no weight on it changes after placing a weight on the Linbot.

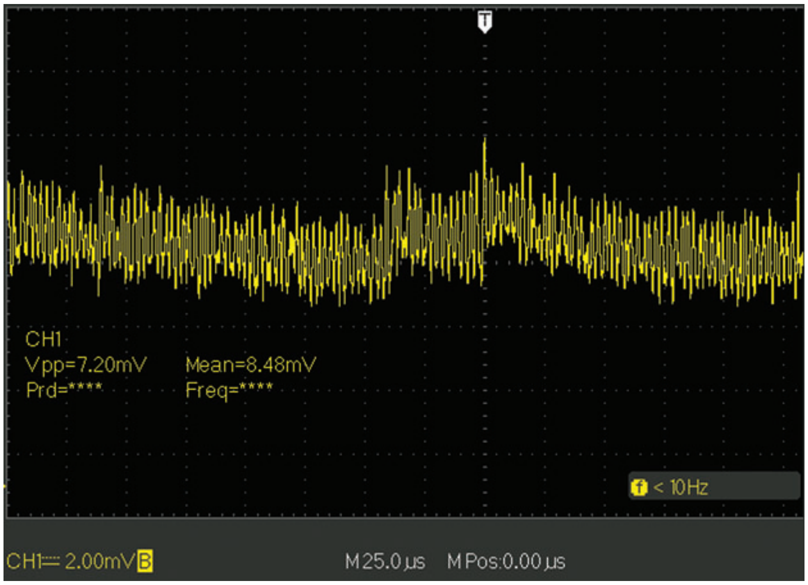

SUPPLEMENTARY FIG. S12. Background noise output from receiver circuit.
