# Supplementary figures and images for: Linbots: Soft Modular Robots Utilizing Voice Coils
Source: Soft Robot. 2019 Apr 16;6(2):195–205. doi: 10.1089/soro.2018.0058 (PMC6486669; doi:10.1089/soro.2018.0058)

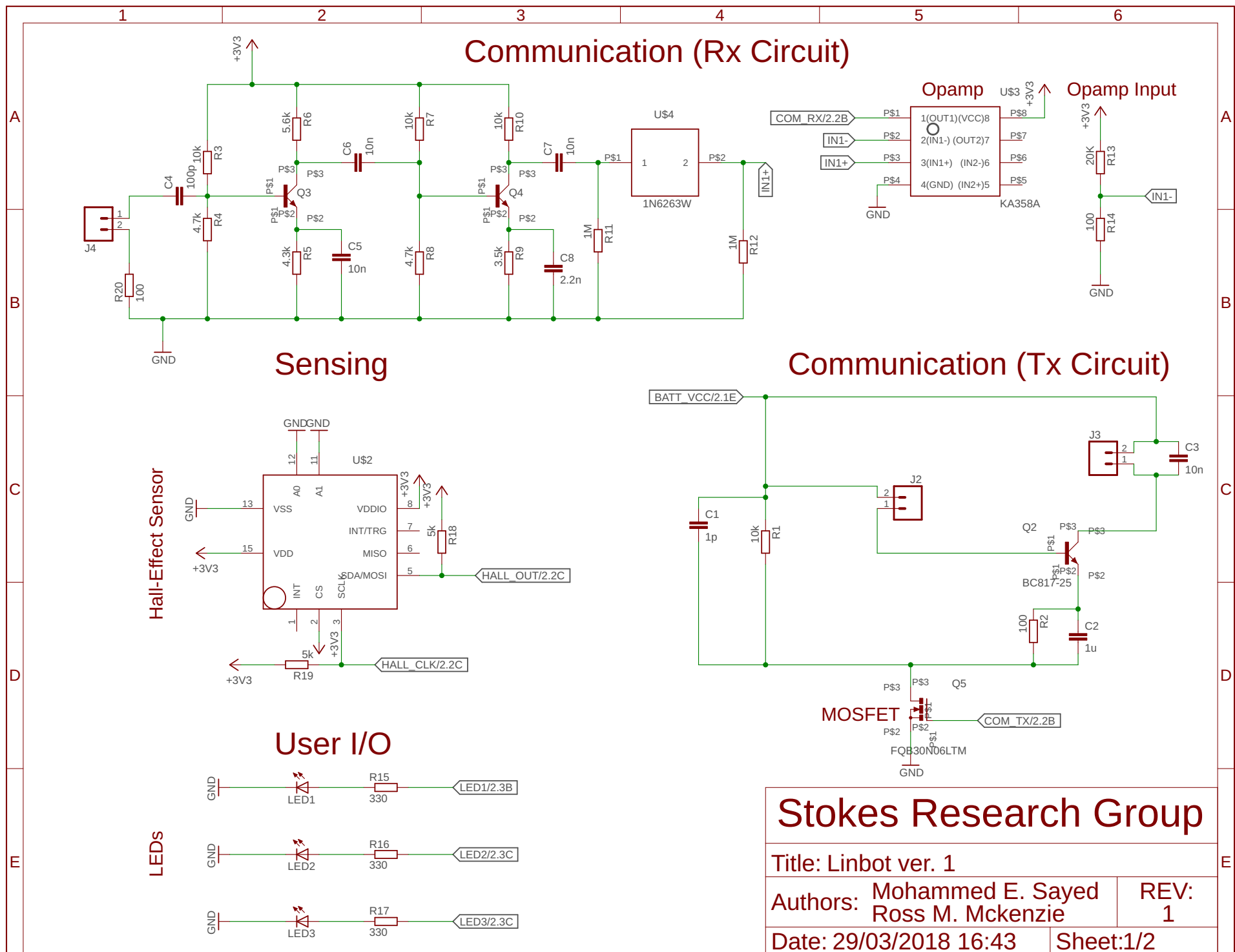

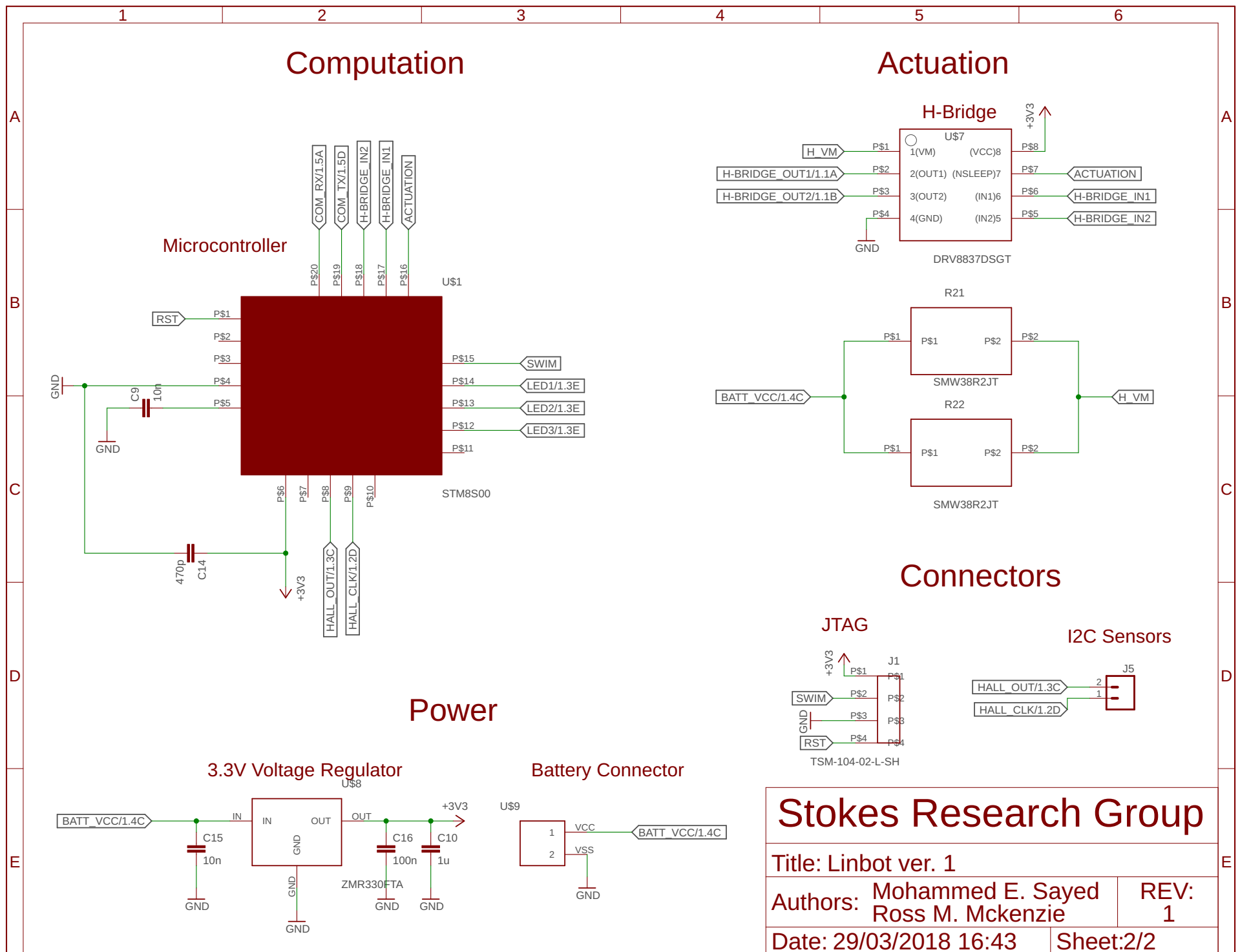

Supplement: Supplemental data [file Supp_Data.zip › PCB Schematic.pdf]
